# A Mechanism of Synergistic Effect of Streptomycin and Cefotaxime on CTX-M-15 Type β-lactamase Producing Strain of *E. cloacae:* A First Report

**DOI:** 10.3389/fmicb.2016.02007

**Published:** 2016-12-15

**Authors:** Lubna Maryam, Asad U. Khan

**Affiliations:** Interdisciplinary Biotechnology Unit, Medical Microbiology and Molecular Biology Laboratory, Aligarh Muslim UniversityAligarh, India

**Keywords:** antibiotic resistance, synergy, beta-lactamase, streptomycin, cefotaxime

## Abstract

A *bla*_CTX-M-15_ gene is one of the most prevalent resistant marker found in member of enterobacteriaceae. It encodes cefotaxime hydrolysing β-lactamase-15 (CTX-M-15) causing resistance against beta lactam antibiotics. Since single antibiotic therapy fails to control infection caused by multidrug resistance strain, therefore combination therapy was came into practice as an effective treatment. We have first time explained the mechanism where two antibiotics of different classes work against resistant strains. Binding parameters obtained by spectroscopic approach showed significant interaction and complex formation between drugs and CTX-M-15 enzyme with decreased k_sv_ and k_q_ values. CD analysis showed altered conformation and significant changes in alpha helical content of CTX-M-15 enzyme on interaction with streptomycin in combination with cephalosporin. Steady state kinetics revealed decrease in hydrolytic efficiency of enzyme to about 27% by cooperative binding behavior upon sequential treatment of enzyme with streptomycin and cefotaxime. Therefore, the study concludes that combination therapy against CTX-M-15 producing strain with Cefotaxime/Streptomycin in 1:10 molar ratio, decreases CTX-M-15 efficiency significantly because of the fact that streptomycin induced structural changes in CTX-M-15 hence cefotaxime was not properly bound on its active site for hydrolysis rather available for the target to inhibit bacterial cells.

## Introduction

β-lactamases are the group of enzymes that cleave amide bond in beta lactam rings of beta lactam antibiotics rendering them harmless to bacteria. Production of these enzymes is the predominant cause of gram negative bacterial resistance against β-lactam antibiotics which has become a major health concern (Bonnet, 2004; Bush, 2010a).

In Enterobacteriaceae, ESBLs (Extended spectrum beta lactamases) encoding CTX-M type markers on plasmid, are reported worldwide (Coque et al., 2008; Hawkey and Jones, 2009). It hydrolyzes the oxyimino-cephalosporin and cefotaxime with about 1000-fold higher catalytic efficiency than other class A β-lactamases (Bauernfeind et al., 1992; Bonnet, 2004). CTX-M type ESBLs display greater hydrolytic activity against Cefotaxime than Ceftazidime (Bonnet, 2004). CTXM-15 is the widely spread ESBL in India and is reported in wide members of Enterobacteriaceae family (Karim et al., 2001). It has been reported that bacteria expressing ESBLs are resistant toward various β-lactam antibiotic groups such as Penicillins, different generations of Cephalosporins, Aztreonam and also to various antibiotic /inhibitor combinations (Faheem et al., 2013). The widespread dissemination of CTX-M-15 by *E. coli* and other enteric bacilli has a significant impact on hospital and community-acquired infections (Bush, 2010b; Chen et al., 2014).

It has been observed that treatment with single antibiotic fails to cure increasing microbial infections due to emergence of antibiotic resistance. For e.g., studies performed on other β-lactamases such as OXA-51 in *Acinetobacter baumannii* shows how carbapenem antibiotic is hydrolyzed by β-lactamases leading to the survival of the pathogen (Tiwari and Moganty, 2014) Therefore, it is an augmented need to employ combination therapy and to understand pharmacological and pharmacodynamic (Lin et al., 1987) behavior of multiple drugs for rational basis of antibiotics selection for effective treatment in order to avoid antagonism between certain antibiotics as demonstrated earlier (Gunnison et al., 1950).

A marked increase in bactericidal effect *in vitro* by synergistic treatment with penicillin and streptomycin has been reported earlier (Jawetz et al., 1951), compared to single drug treatment (Gunnison et al., 1950). An observation which is consistent with a hypothesis that streptomycin faces natural barrier while entering enterococci which can be prevailed by agents inhibiting cell wall synthesis such as penicillin and hence can produce synergistic effect (Moellering and Weinberg, 1971). We have already reported earlier that the synergistic effect of cefoxitin with streptomycin and cefotaxime proved an effective combination against multidrug resistant bacterial strains (Hasan et al., 2013).

The mechanism behind effective nature of drugs of two different classes in combination has not yet been explained. Therefore, this is the first time we have initiated this work to understand the molecular mechanism behind synergy of cephalosporin and aminoglycoside against multidrug resistance strains. The hypothesis proposed was that streptomycin might induce structural changes in CTX-M-15 enzyme on binding, hence may not allow cefotaxime to properly bind and hydrolyze, as a result cefotaxime is available for target site inhibition in bacterial cells.

## Materials and methods

### Protein/enzyme source

CTX-M-15 from *Enterobacter cloacae* clinical strain, EC-15 (Genebank accession no.: JN860195.1) (Chen et al., 2014) *E. coli* BL21 (DE3), pQE-2 (high copy cloning vector).

### Antibiotics and other chemicals

Cefotaxime and Cefoxitin were purchased from Sigma chemical co. (St. Louis, MO), Streptomycin from Himedia (India), IPTG (isopropyl-β-D-1-thiogalactopyranoside) was purchased from Roche (Basel, Switzerland). Nitrocefin was purchased from Calbiochem (USA). Imidazole was purchased from Sigma-Aldrich. Other reagents and chemicals were of analytical grade and double distilled water was used throughout the study.

### *bla*_CTX-M-15_ cloning and expression of CTX-M-15

The plasmid DNA carrying *bla*_CTXM-15_ gene cloned from clinical *E. cloacae* EC-15 strain (Genebank accession no.: JN860195.1), was extracted using Qiagen plasmid extraction kit, according to manufacturer's instructions. The *bla*_CTX-M-15_ was amplified by PCR using primers CTX-M-15-F (5′ ATATCATATGGTTAAAAAATCACTG 3′) containing Nde I restriction site and CTX-M-15-R (5′ ATATAAGCTTTTACAAACCGTCGGTGAC 3′) containing Hind III restriction site. The PCR conditions used were 95°C for 30 s, 54°C for 25 s, 72°C for 40 s and the reaction process was carried out for 35 cycles (Faheem et al., 2013). The PCR product does not contain the promoter region of the gene. The PCR product and pQE-2 (high copy cloning vector), were double digested with NdeI and Hind III endonucleases, ligated and transformed into competent *E. coli* BL21 (DE3) cells by heat shock method (4°C for 30 min, 42°C for 90 s and 4°C for 10 min). Transformants harboring *bla*_CTX-M-15_ gene were selected on LB agar plates containing ampicillin (100 μg/ml). The clones were confirmed by double restriction digestion of obtained transformed cells by NdeI and HindIII enzymes (Figure S1) and sequencing by standard procedures.

To express and purify CTXM-15 β-lactamase, the competent cells of *E. coli* BL21 (DE3) harboring pQE-2 vector carrying *bla*_CTX-M-15_ gene, a 10 ml overnight culture of these transformed cells in Luria-Bertani broth containing 100 μg/ml ampicillin was used to inoculate 1 l of Luria-Bertani broth containing 100 μg/ml ampicillin. Bacterial culture was grown at 37°C with shaking at 120 rpm, until an optical density of 0.6–0.8 was reached at 600 nm (Faheem et al., 2013). The culture was cooled and induced with 0.2 mM IPTG and placed at 16°C at 150 rpm for 12–16 h. The bacterial cells were collected by centrifugation and re-suspended in 20 ml lysis buffer containing 50 mM Sodium phosphate (pH 8.0), 300 mM NaCl and 10 mM Imidazole along with 0.1% β-mercaptoethanol per liter culture. The bacterial cells were ruptured by sonication at 35% amplitude for 10 min, and the cell debris obtained was removed by centrifugation at 12,000 rpm for 30 min. The clear supernatant was loaded onto a Ni-NTA column, which was pre-equilibrated by lysis buffer, and washed with lysis buffer supplemented with 50 mM imidazole. Protein was eluted with PBS (Phosphate buffer saline, 50 mM sodium phosphate (pH 8.0) containing 300 mM NaCl) buffer containing 250 mM imidazole. Pure protein was obtained after dialysis in PBS. Purity of the purified protein was estimated to be more than 97% as determined by a single band of 31 kDa on SDS-PAGE (Figure S2). The final protein concentrations were determined by using the molar extinction coefficient of 25, 440 M^−1^ cm^−1^ at 280 nm.

### Fluorescence spectra measurements

All the fluorescence study was done on a Shimadzu RF-5301PC spectrofluorometer (Shimadzu Corporation, Kyoto, Japan) which is equipped with a thermostatically controlled cell holder and attached to a water bath to maintain desired constant temperature. Fluorescence quenching was monitored by measuring intrinsic fluorescence quenching of protein to elucidate the mechanism of its interaction with drug molecule (Eftink and Ghiron, 1976; Lakowicz, 1988) between 300 and 450 nm after selectively exciting the sample at 295 nm, both the excitation and emission slits were set at 5 nm and the spectra were recorded at fast scanning mode. To a 3 mL sample containing 2 μM CTX-M-15 protein alone or incubated with 20 μM of cefotaxime, cefoxitin and streptomycin each in various combinations and by successive direct addition of 2 μM of each drug in such a manner that the total volume added was not more than 40 μL at 298 K. All the fluorescence intensities were corrected for the inner filter effect. The decrease in fluorescence intensity of protein at emission maxima was analyzed by using the Stern-Volmer equation (Lakowicz, 1988):

(1) $$\begin{array}{l} \text{F}{}^{\circ}/\text{F}=\;1+\text{ Ksv}\left[\text{Q}\right]=1+\text{ Kq}\tau {}^{\circ}\left[Q\right] \end{array}$$

where F° and F are the fluorescence intensities in the absence and presence of drug (quencher), K_SV_ is the Stern-Volmer constant, [Q] is the molar concentration of quencher, and kq and τ° are the bimolecular quenching rate constant and the lifetime of the protein fluorescence in the absence of quencher, respectively. The bimolecular rate constant Kq was calculated from the relation:

(2) $$\begin{array}{l} \text{Kq}=\text{Ksv}/\tau {}^{\circ} \end{array}$$

where τ° is the mean fluorescence life time of Trp which is ~ 4.31 × 10^−9^ s. The binding constant (Ka) and the number of binding sites (n) were calculated using the following modified Stern-Volmer equation (Kang et al., 2004):

(3) $$\begin{array}{l} \text{log}\frac{\text{F}{}^{\circ}-\text{F}}{\text{F}}=\text{logKa}+\text{nlog}\left[\text{Q}\right] \end{array}$$

### CD spectra measurements

CD spectra were collected on a Jasco J-810 spectropolarimeter (Jasco International Co. Ltd., Tokyo, Japan) equipped with a Peltier-type temperature controller (PTC-423S/15) and attached to a water bath. Far-UV CD spectra of CTX-M-15 in the absence and presence of drugs (1:10) (Micro molar ratio) were taken at protein concentrations of 5 μM in 0.1 cm path length cells, respectively and all the spectra were corrected for the appropriate blanks. The instrument was calibrated with (+)-10-camphorsulfonic acid. All the spectra were measured at 298 K using a scan speed of 100 nm/min and the response time of 1 s. The observed ellipticity obtained is converted to mean residual ellipticity [MRE] in deg.cm^2^.dmol^−1^ by using the following equation (Rehman et al., 2015):

(4) $$\begin{array}{l} \text{MRE}=\frac{\left[\theta \right]\text{obs}}{10\text{ncl}} \end{array}$$

Where [θ]obs is the observed ellipticity in mdeg, n is the total number of amino acid residues (291) in CTX-M-15 protein, c is the molar concentration of the protein, and l is the path length in cm. The α-Helical content of drug treated and untreated CTX-M-15 was calculated from the MRE values at 208 and 222 nm using the following equation (Chen et al., 1972):

(5) $$\begin{array}{l} \%\alpha -\text{helix}=\left[\frac{\left[\text{MRE}\right]208\text{nm}-4000}{33000-4000}\right]*100 \end{array}$$

(6) $$\begin{array}{l} \%\propto -\text{helix}=\left[\frac{\left[\text{MRE}\right]222\text{nm}-2340}{30300}\right]*100 \end{array}$$

### Steady-state kinetics experiments

Enzyme kinetics measurements were recorded on a Shimadzu UV-1800 double beam spectrophotometer (Shimadzu International Co. Ltd., Kyoto, Japan) at 298 K.

The hydrolysis activity of CTX-M-15 toward a chromogenic cephalosporin substrate Nitrocefin was studied (O'Callaghan et al., 1972). Steady-state enzyme kinetics was performed by directly monitoring the initial velocities of appearance or disappearance of chromophore, Nitrocefin. The effect of Cefotaxime, Streptomycin, and Cefoxitin binding on the catalytic activity of CTX-M-15 toward Nitrocefin was determined by steady state kinetics at pH 7.4 in 50 mM phosphate buffer and 298 K. The concentration of CTX-M-15 enzyme was kept constant at 8.75 nM, (for dilution of the enzyme and to prevent denaturation, BSA was added to a final concentration of 20 μg/ml (Galleni et al., 1994), we found that BSA at the concentration used in the experiment did not show any effect on the hydrolytic ability of CTX-M-15), while the concentration of nitrocefin was varied from 0 to 650 μM. CTX-M-15 activity in presence of 87.5 nM of each drug, cefotaxime, streptomycin and cefoxitin was obtained by incubating them for 1.5 h with enzyme at room temperature. The rate of Nitrocefin hydrolysis was determined by measuring the appearance of nitrocefin (red colored product) at 486 nm for 65 s. All the measurements were performed on Shimadzu UV-1800 double beam spectrophotometer. The concentration of nitrocefin was determined by measuring absorbance using a molar extinction coefficient value of 15,000 M^−1^cm^−1^ at 486 nm. The kinetic parameters (kcat and Km) were determined according to the Michaelis-Menten method by fitting the data to the following equations.

(7) $$\begin{array}{l} \text{v}=\frac{\text{Vmax}\left[\text{S}\right]}{\text{Km}+\left[\text{S}\right]} \end{array}$$

(8) $$\begin{array}{l} \text{Kcat}=\frac{\text{Vmax}}{\left[\text{E}\right]} \end{array}$$

## Results

The *bla*_CTX-M-15_ gene was cloned and transformants harboring *bla*_CTX-M-15_ gene were confirmed by double restriction digestion using NdeI and HindIII enzymes for its presence. Agarose gel of double digestion showed two bands of 4.8 kb corresponding to pQE-2 vector and 800 bp corresponding to *bla*_CTX-M-15_ gene (Figure S1). Purity of the protein obtained after dialysis in PBS was estimated to be more than 97% as determined by single band of 31 kDa on SDS-PAGE (Figure S2). The final protein concentrations were determined to be 1.5 mg/ml using the molar extinction coefficient of 25, 440 M^−1^ cm^−1^ at 280 nm.

**Figure 1:**
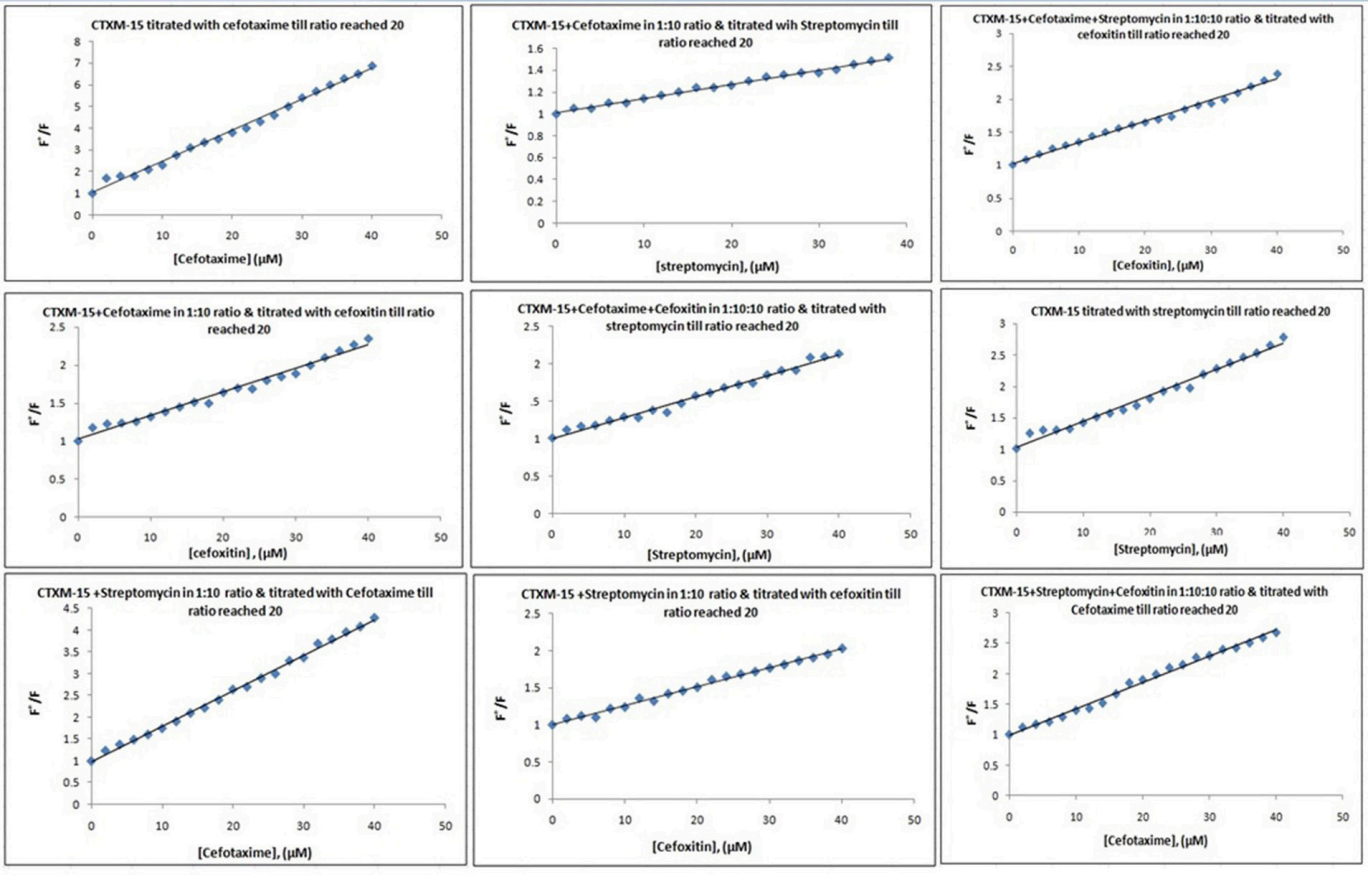
**Stern-Volmer plots for drug induced fluorescence quenching of CTX-M-15**. At 298 K under different drug incubations, the Stern-Volmer plots for CTX-M-15- drug interactions is shown. The concentration of CTX-M-15 was 2 μM and the concentration of the bounded drug was 20 μM in 50 mM sodium phosphate buffer at pH 7.4.

**Figure 2:**
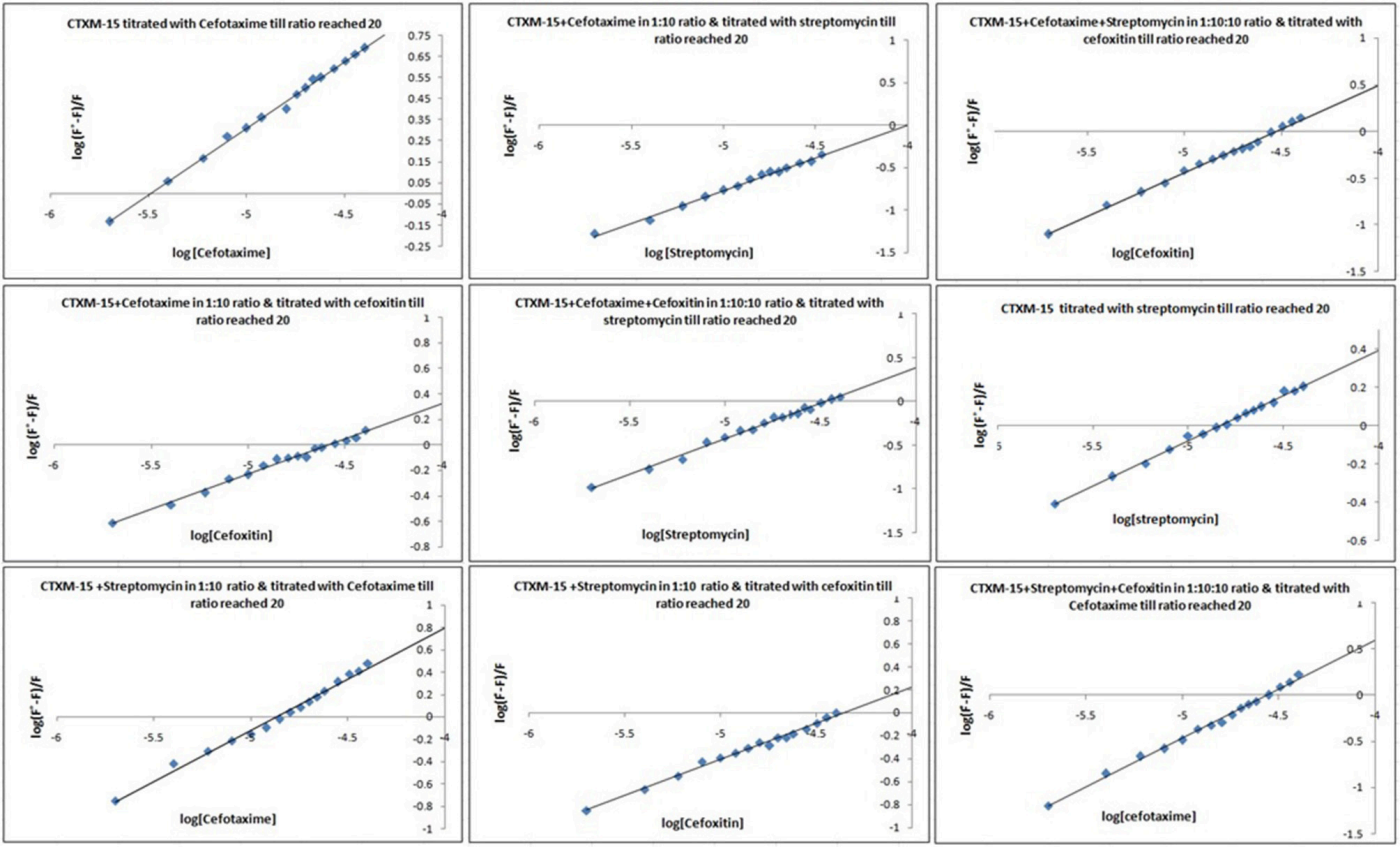
**Modified Stern−Volmer plots for the quenching of CTX-M-15**. At 298 K under different drug incubations, the Modified Stern-Volmer plots for CTX-M-15- drug interactions is shown. The concentration of CTX-M-15 was 2 μM and the concentration of the bounded drug was 20 μM in 50 mM sodium phosphate buffer at pH 7.4.

Fluorescence spectra measurements showed the effect of single and combination of drug binding on the fluorescence property of CTX-M-15 (Figures S3, S4). A progressive decrease in the fluorescence intensity was observed due to quenching of CTX-M-15 fluorescence. The data were analyzed according to the Stern-Volmer Equations (1) and (2) (Figure 1, Table 1). The binding constant (Ka) lying in the range of 10^2^–10^4^ M^−1^ and the number of binding sites (n) which was found to be one, were determined using modified Stern-Volmer Equation (3) (Figure 2, Table 1). K_*SV*_ values for interactions with different drugs were of the order of 10^4^ M^−1^. The kq values were determined from the ratios of K_SV_/τo after taking τo for CTX-M-15 = 4.31 × 10^−9^s. The kq values were of the order of 10^12^–10^13^ M^−1^s^−1^.

**Table 1:** **Stern−Volmer quenching constants and binding parameters for CTX-M-15 and Cefotaxime/Streptomycin/Cefoxitin interactions**.

	**Ksv(M^−1^)**	**Kq(M^−1^s^−1^)**	**Ka(M^−1^)**	**n**	**R^2^**
CTX-M-15+CTX	14.6 × 10^4^	3.38 × 10^13^	0.297 × 10^4^	0.633	0.995
CTX-M-15+CTX+FOX	3.2 × 10^4^	0.74 × 10^13^	0.037 × 10^4^	0.558	0.993
CTX-M-15+CTX+FOX+STR	2.8 × 10^4^	0.64 × 10^13^	0.361 × 10^4^	0.794	0.991
CTX-M-15+CTX+STR	1.2 × 10^4^	0.278 × 10^13^	0.126 × 10^4^	0.773	0.994
CTX-M-15+CTX+STR+FOX	3.2 × 10^4^	0.742 × 10^13^	1.253 × 10^4^	0.904	0.991
CTX-M-15+STR	4.4 × 10^4^	1.02 × 10^13^	0.019 × 10^4^	0.47	0.997
CTX-M-15+STR+CTX	8.2 × 10^4^	1.902 × 10^13^	2.904 × 10^4^	0.916	0.993
CTX-M-15+STR+FOX	2.5 × 10^4^	0.58 × 10^13^	0.162 × 10^4^	0.729	0.992
CTX-M-15+STR+FOX+CTX	4.2 × 10^4^	0.974 × 10^13^	2.023 × 10^4^	0.944	0.994

*CTX, cefotaxime; STR, streptomycin; FOX, cefoxitin*.

**Figure 3:**
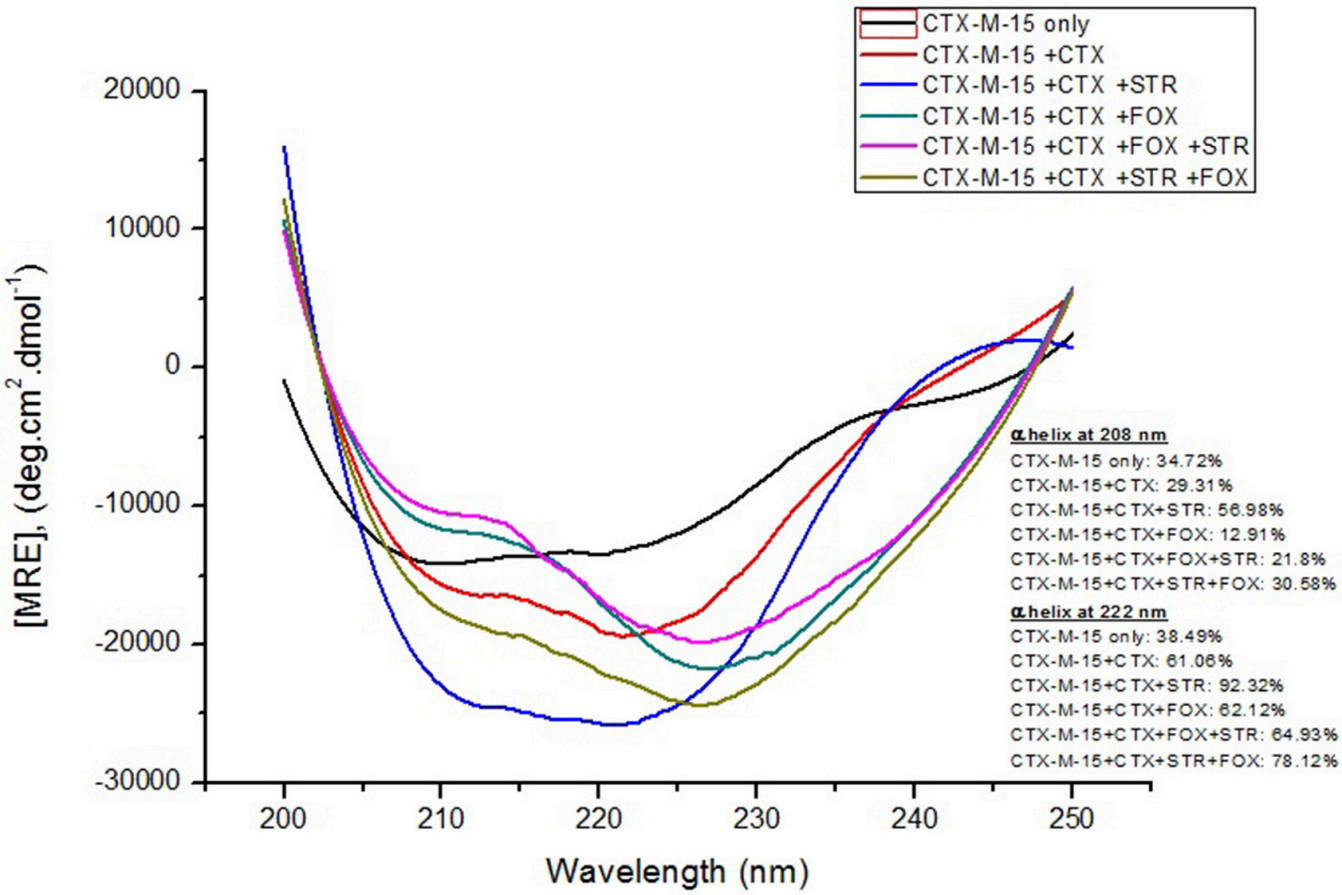
**Far-UV CD spectra of CTX-M-15**. CD spectra of CTX-M-15 alone or in complex with cefotaxime alone or along with streptomycin/cefoxitin or both at 1:10, 1:10:10, and 1:10:10:10 molar ratio was taken. The concentration of CTX-M-15 and cefotaxime/streptomycin/cefoxitin was 5 and 50 μM in 50 mM Sodium phosphate buffer pH 7.4 at 298 K.

**Figure 4:**
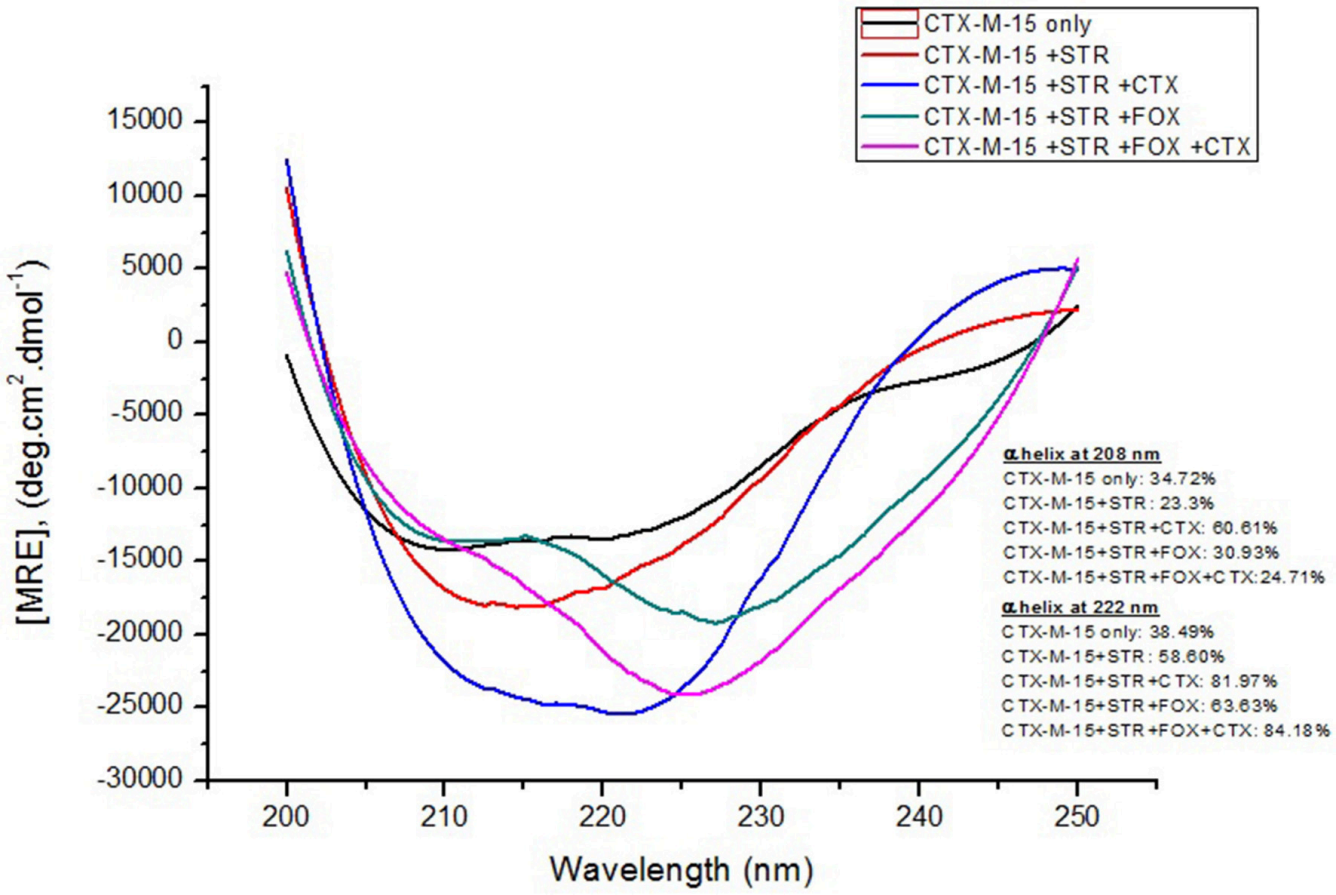
**Far-UV CD spectra of CTX-M-15**. CD spectra of CTX-M-15 alone or in complex with streptomycin alone or along with cefotaxime/cefoxitin or both at 1:10, 1:10:10, and 1:10:10:10 molar ratio was taken. The concentration of CTX-M-15 and streptomycin/cefotaxime/ cefoxitin was 5 and 50 μM in 50 mM Sodium phosphate buffer pH 7.4 at 298 K.

Far-UV CD spectrum characterizes the conformation of the peptide backbone to determine secondary structure (α-helices and β-sheets) of the protein. Hence the possible effects of (single/combination) drug on the secondary structure of CTX-M-15 was monitored by CD spectroscopy in the far-UV region (250–200) and the results are shown in Figures 3, 4. CTX-M-15, in the absence of drug showed two negative bands at 208 and 222 nm which is a characteristic of the α-helix protein with minima at 208 and 222 nm. The far-UV CD spectra of CTX-M-15 closely resembled to that of the CTX-M-1 (Perez-Llarena et al., 2011). MRE (mean residual ellipticity) and % alpha helical content of the protein calculated are shown in Table 2. The MRE_208_ nm and MRE_222_ nm of CTX-M-15 under native condition, without any drug treatment was found to be −14,070 and −14,004.9 deg cm^2^ dmol^−1^ respectively (Table 2), with alpha helical content of 34.72 and 38.49% as calculated from Equations (5) and (6). In the presence of drug combinations, CTX-M-15 showed remarkable distortion in the alpha helical content with maximum disruption in streptomycin and cefotaxime bounded CTX-M-15.

**Table 2:** **Spectral characteristics of CTX-M-15 under different drug binding conditions**.

	**MRE_208_ (deg.cm^2^.dmol^−1^)**	**% α helix at 208**	**MRE_222_ (deg.cm^2^.dmol^−1^)**	**%** α **helix at 222**
CTX-M-15	−14,070 ± 210	34.72%	−14,004.9 ± 145	38.49%
CTX-M-15+CTX	−12,500.1 ± 161	29.31%	−20,842.9 ± 132	61.06%
CTX-M-15+CTX+STR	−20,524.3 ± 170	56.98%	−30,313.2 ± 126	92.32%
CTX-M-15+CTX+FOX	−7,744.89 ± 157	12.91%	−21,163.5 ± 201	62.12%
CTX-M-15+CTX+FOX+STR	−10,322.8 ± 190	21.8%	−22,061.1 ± 138	64.93%
CTX-M-15+CTX+STR+FOX	−12,871 ± 173	30.58%	−26,010.9 ± 223	78.12%
CTX-M-15+STR	−10,760.6 ± 102	23.3%	−20,096.1 ± 139	58.60%
CTX-M-15+STR+CTX	−21,577.9 ± 189	60.61%	−27,178.1 ± 159	81.97%
CTX-M-15STR+FOX	−12,972.1 ± 144	30.93%	−21,621.4 ± 149	63.63%
CTX-M-15+STR+FOX+CTX	−11,166.3 ± 204	24.71%	−27,848.7 ± 187	84.81%

*CTX, cefotaxime; STR, streptomycin; FOX, cefoxitin*.

*MRE values are reported as the average of ± standard error from three independent experiment*.

**Figure 5:**
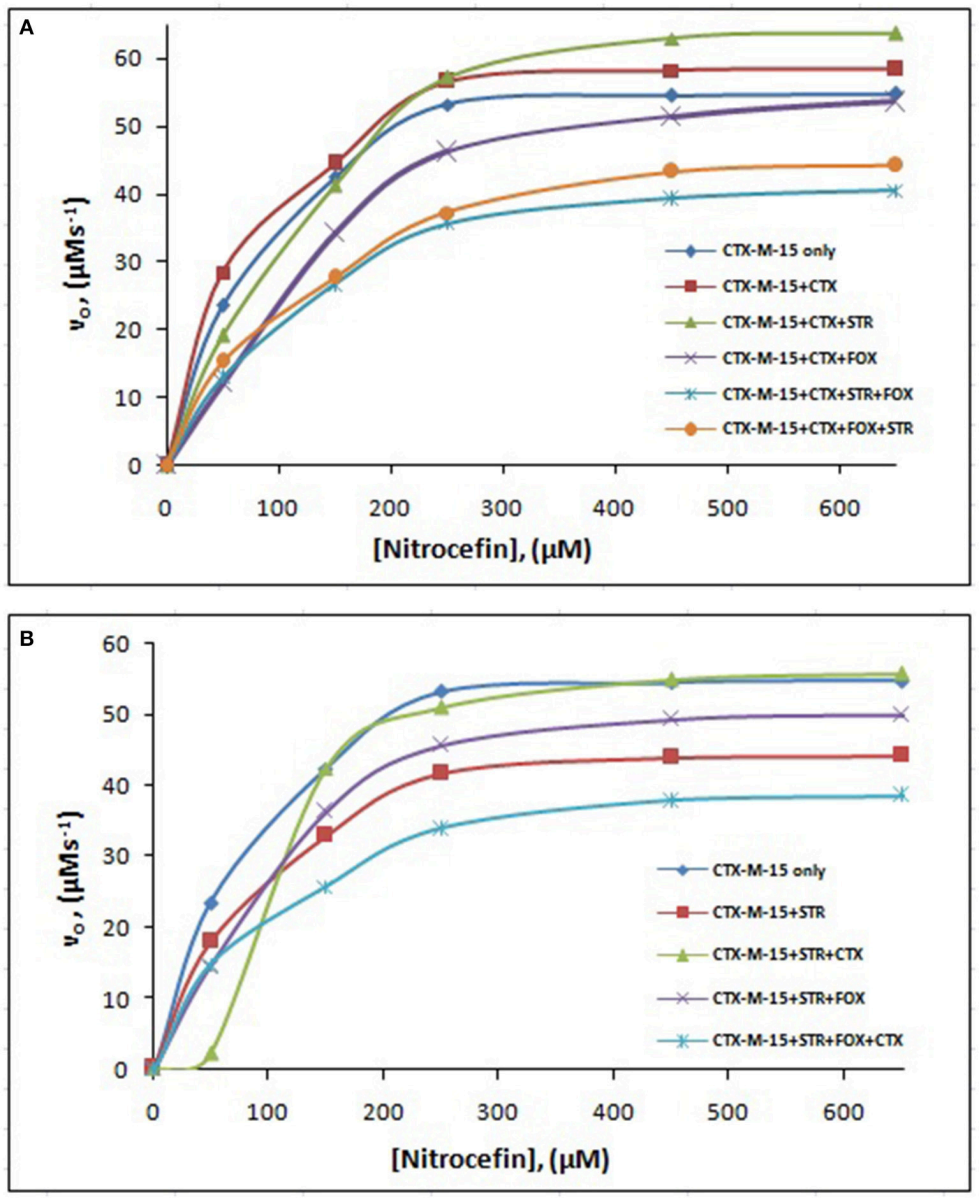
**Michaelis-Menten plot**. Steady-state kinetics of Nitrocefin hydrolysis by CTX-M-15 in the absence and presence of different combinations of **(A)** Cefotaxime/Streptomycin/Cefoxitin and **(B)** Streptomycin/ Cefotaxime/Cefoxitin at 298 K was carried out. The concentration of CTX-M-15 was 8.75 nM while the concentration of drugs were 87.5 nM in 50 mM sodium phosphate buffer, pH 7.4.

**Figure 6:**
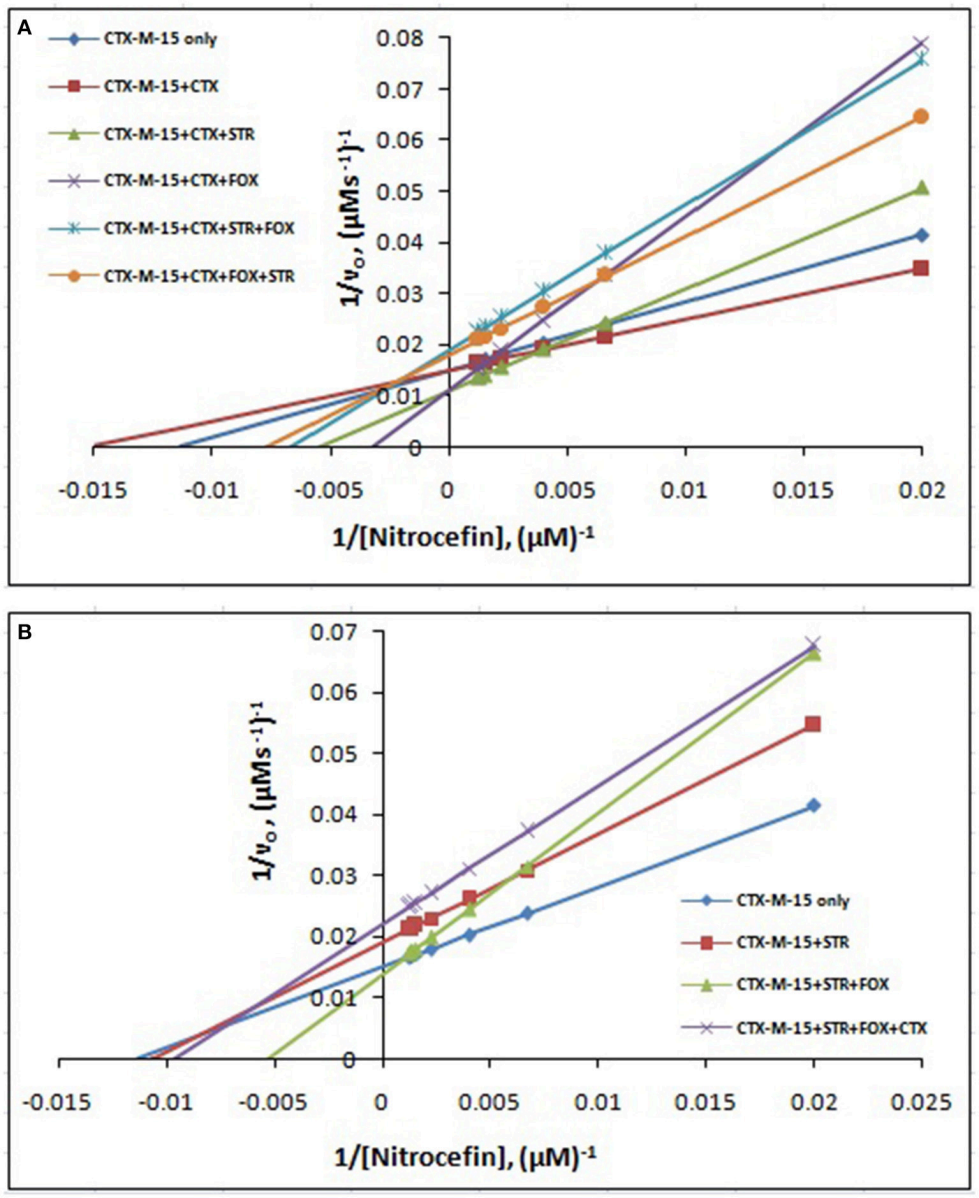
**Lineweaver-Burk plot**. Steady-state kinetics of Nitrocefin hydrolysis by CTX-M-15 in the absence and presence of different combinations of **(A)** Cefotaxime/Streptomycin/Cefoxitin and **(B)** Streptomycin/ Cefotaxime/Cefoxitin at 298 K was carried out. The concentration of CTX-M-15 was 8.75 nM while the concentration of drugs were 87.5 nM in 50 mM sodium phosphate buffer, pH 7.4.

The steady-state kinetics of the purified CTX-M-15 was carried out on nitrocefin, a chromogenic cephalosporin substrate which revealed a hydrolytic profile that is a characteristic of molecular class A beta lactamase. The representative Michaelis-Menten plots are shown in Figure 5 and the deduced kinetic parameters (kcat, Km, and kcat/Km) are summarized in Table 3. The catalytic activity of CTX-M-15 was investigated on nitrocefin to ascertain the involvement of CTX-M-15 in drug binding. The Michaelis-Menten plots of nitrocefin hydrolysis at 1:10, 1:10:10, 1:10:10:10 molar ratios of different CTX-M-15: cefotaxime/streptomycin/cefoxitin, drug combinations were analyzed to check if kcat and Km values in any case being reduced. The kinetic data were also plotted as Lineweaver-Burk plots to infer the kinetic parameters by which drug inhibited the hydrolysis activity of CTX-M-15 (Figure 6). We found increased Km values for all CTX-M-15 drug incubations except for cefotaxime alone with CTX-M-15, implying the decrease in the affinity of enzyme toward substrate nitrocefin in the presence of streptomycin and in all studied combination of streptomycin and cephalosporin drugs treatment. Similarly we found decreased catalytic efficiency (Kcat/Km) of enzyme for all streptomycin and cephalosporin combinations except for cefotaxime alone. Calatytic activity (Kcat) of the enzyme with nitrocefin was found to decrease in all combinations except in presence of cefotaxime, cefotaxime along with streptomycin and cefoxitinand streptomycin along with cefoxitin.

**Table 3:** **Steady-State Kinetic Parameters for Hydrolysis activity of CTX-M-15 in presence of various drugs**.

	**Km (μM)**	**Kcat (s^−1^)**	**Kcat/Km (μM^−1^s^−1^)**
CTX-M-15 only	88.13 ± 0.5	7618.97 ± 1.4	86.451
CTX-M-15+ CTX	65.932 ± 0.9	7618.97 ± 0.85	115.557
CTX-M-15+ STR	93.789 ± 1.0	6015.02 ± 0.3	64.133
CTX-M-15+STR+CTX (Allosteric curve)	~ 100 ± 2.0	~ 6285 ± 3.1	~ 62.85
CTX-M-15+STR+FOX	187.212 ± 1.6	8163.2 ± 2.5	43.604
CTX-M-15+CTX+STR	180.363 ± 1.8	10389.6 ± 0.4	57.603
CTX-M-15+CTX+FOX	304.45 ± 0.9	10389.6 ± 2.2	34.12
CTX-M15+STR+FOX+CTX	103.589 ± 0.6	5197.74 ± 1.3	50.176
CTX-M-15+CTX+STR+FOX	149.419 ± 0.2	6014.971 ± 0.7	40.255
CTX-M-15+CTX+FOX+STR	129.165 ± 2.1	6349.142 ± 1.5	49.155

*CTX, cefotaxime; STR, streptomycin; FOX, cefoxitin*.

*Kinetic constants are reported as the average of ± standard error from three independent experiment*.

## Discussion

Fluorescence quenching phenomenon occurs due to various molecular interactions such as reaction in the excited state when the electrons in the higher energy level is returned back to lower energy level, energy transferring, molecular rearrangements and static and dynamic quenching. It is being carried out to elucidate the mechanism of interaction of enzyme with drug molecules (Eftink and Ghiron, 1976; Lakowicz, 1988). Fluorescence quenching spectra showed the linear dependence of quenching with different drug combinations which implies that only one type of quenching mechanism either static or dynamic dominated in the process (Rehman et al., 2014). There is significant interaction between CTX-M-15 and drugs (cefotaxime, cefoxitin, and streptomycin), which was responsible for the quenching mechanism, inferred by Ksv values (~ 10^4^ M^−1^) as shown in Table 1. The kq values in all the cases were of the order of 10^12^–10^13^ M^−1^s^−1^ which were found considerably larger than the maximum dynamic quenching constant ~ 10^10^ M^−1^s^−1^, indicating that the drug-induced quenching of CTX-M-15 fluorescence was due to complex formation. The microenvironment around the binding site is becoming less hydrophobic upon binding of drug by exposing more residues for interaction as seen by increased values of binding sites.

Greater interaction of CTX-M-15 with cefotaxime than streptomycin was observed with higher K_SV_ (stern volmer constant), kq (binding constant) and Ka (association constant). Decrease in K_SV_ and kq was observed in all the combinations of drug binding with respect to cefotaxime, suggesting that the quenching of CTX-M-15 fluorescence was initiated by complex formation in the ground state rather than by dynamic quenching. However, increase in the value (n) in all the cases except when CTX-M-15 interacted with cephalosporins or aminoglycoside, shows that the microenvironment around the binding site is becoming less hydrophobic upon binding of drug. It is probably due to exposure of more binding site residues for interaction. Maximum drug interaction was observed in all the three triple combinations of drug and combination of streptomycin and cefotaxime with CTX-M-15 with the increase in the values of binding constant comparing to CTX-M-15 with cefotaxime alone. It indicates that streptomycin induces structural changes in CTX-M-15 thereby making conditions unfavorable for proper binding of cefotaxime on its active site for hydrolysis. Hence decrease in stern volmer and quenching constant was observed.

Far-UV CD spectra showed remarkable change in secondary structure of CTX-M-15 enzyme at 208 and 222 nm with respect to native enzyme in all the drug treated combinations. At 208 nm all the combinations showed at least 3.7% decrease in alpha helix peak. Whereas, on combination with cefotaxime and streptomycin, 22.2% increase in the peak was observed. At 222 nm all the combinations showed 24.11% increase in the peak. While, cefotaxime and streptomycin in combination showed maximum rise in the peak (47.48%) as shown in Figures 3, 4. Hence the CD-spectral analysis showed disruption in the overall conformation of CTX-M-15 enzyme upon both single and combined binding of cephalosporin and aminoglycoside. Moreover, conformational changes upon binding of cefotaxime favors hydrolysis, whereas structural changes occurred due to binding in combination, leading to reduced hydrolysis of cefotaxime.

**Figure 7:**
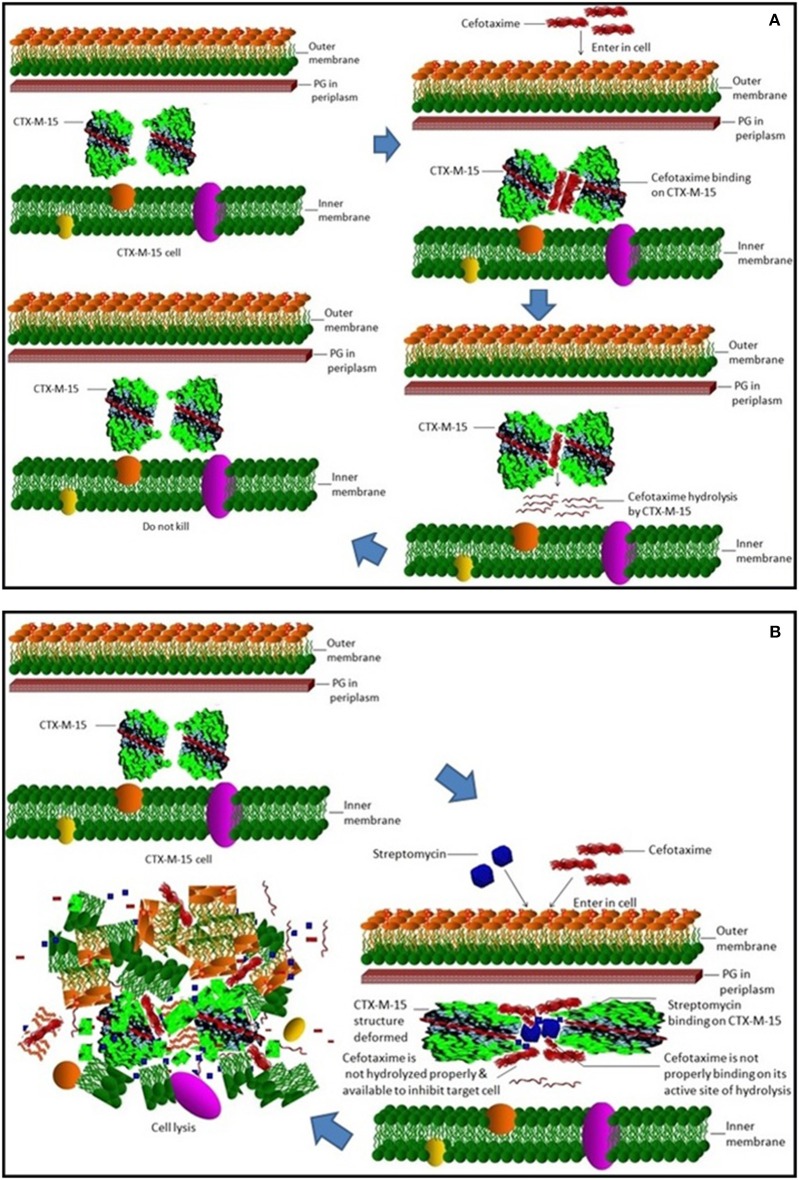
**Synergistic effect of streptomycin and cefotaxime on CTX-M-15**. **(A)** shows inability of cell killing by cefotaxime due to its hydrolysis by CTX-M-15 enzyme. **(B)** shows cellular lyses by available un-hydrolyzed cefotaxime due to streptomycin which leads to reduced binding of cefotaxime on its active site of hydrolysis.

Steady-state kinetics data showed the decrease in the affinity of the CTX-M-15 enzyme toward nitrocefin in the presence of streptomycin alone and with all other synergistic drug combinations. Also synergistic drug treatment (in 1:10, 1:10:10, 1:10:10:10 molar ratio of CTXM-15 and drug) decreased the catalytic efficiency of enzyme to about 27% in all the cases except in the presence of cefotaxime alone. Enzyme treated with cefotaxime alone was found 33% more efficient, when combined along with streptomycin and cefoxitin, the efficiency was reduced to about 53.4%. Whereas, enzyme treated with streptomycin alone caused to reduce its efficiency by 25.8%, however when combined with cefotaxime, its efficiency was decreased by 27.2% with cooperative sigmoidal binding curve (allosteric behavior). If enzyme combined with cefoxitin, its efficiency was decreased to about 49.5% while in combination of cefoxitin and cefotaxime, it was reduced by 41.9%. Hence, the study shows that the enzyme incubated first with streptomycin then with cefotaxime in 1:10:10 molar ratio, shows allosteric behavior. It indicates that streptomycin binding on enzyme promotes cooperative binding of cefotaxime. Moreover, upon streptomycin incubation as single and in cefotaxime/cefoxitin/streptomycin combinations, catalytic efficiency of enzyme was decreased significantly which supports synergistic effect of two drugs. While single drug was not effective against multidrug resistant strain carrying CTX-M-15 enzyme. This can be explained as binding of streptomycin may induce structural changes in CTX-M-15, hence cefotaxime was not hydrolyzed and available to act on its target in bacterial cells to kill. This is the first time we have demonstrated a possible mechanism of synergistic effect of cefoxitin, streptomycin and cefotaxime against multi-drug resistant strains. In this mechanism based study, streptomycin induced conformational changes in CTX-M-15 leading to reduced binding of cefotaxime on its active site of hydrolysis which in turn decreases its hydrolysis. Consequently, cefotaxime is available for target site inhibition in bacterial cells (Figure 7).

## Conclusion

Our study demonstrated first time a possible molecular mechanism of synergistic effect of combination therapy of streptomycin and cefotaxime against CTX-M-15 producing multi-drug resistant strain. The study revealed that binding of streptomycin induces conformational changes in CTX-M-15 leading to reduced binding of cefotaxime which in turn reduces its hydrolysis. Consequently, cefotaxime is available for target site inhibition in bacterial cells.

## Author contributions

LM: performed experiment and written first draft. AK: designed problem, interpret data, provide reagents, checked manuscript.

## Funding

The work was supported by these grants: DBT GRANT NO. BT/PR8281/BID/7/448/2013, DBT Award NO. BT/HRD/NBA/34/01/2012.

### Conflict of interest statement

The authors declare that the research was conducted in the absence of any commercial or financial relationships that could be construed as a potential conflict of interest.

## Abbreviations

ESBLs: Extended spectrum beta lactamases
IPTG: Isopropyl-β-D-1-thiogalactopyranoside
PBS: phosphate buffer saline
CD: Circular Dichroism.

## References

[B1] Bauernfeind A. Casellas J. M. Goldberg M. Holley M. Jungwirth R. Mangold P.. (1992). A new plasmidic cefotaximase from patients infected with Salmonella typhimurium. Infection 20, 158–163. 10.1007/BF01704610 1644493

[B2] Bonnet R. (2004). Growing group of extended-spectrum beta-lactamases: the CTX-M enzymes. Antimicrob. Agents Chemother. 48, 1–14. 10.1128/AAC.48.1.1-14.2004 14693512 PMC310187

[B3] Bush K. (2010a). Alarming beta-lactamase-mediated resistance in multidrug-resistant Enterobacteriaceae. Curr. Opin. Microbiol. 13, 558–564. 10.1016/j.mib.2010.09.006 20920882

[B4] Bush K. (2010b). Bench-to-bedside review: the role of beta-lactamases in antibiotic-resistant Gram-negative infections. Crit. Care 14, 224. 10.1186/cc8892 20594363 PMC2911681

[B5] Chen L. F. Freeman J. T. Nicholson B. Keiger A. Lancaster S. Joyce M.. (2014). Widespread dissemination of CTX-M-15 genotype extended-spectrum-beta-lactamase-producing enterobacteriaceae among patients presenting to community hospitals in the southeastern United States. Antimicrob. Agents Chemother. 58, 1200–1202. 10.1128/AAC.01099-13 24247126 PMC3910860

[B6] Chen Y. H. Yang J. T. Martinez H. M. (1972). Determination of the secondary structures of proteins by circular dichroism and optical rotatory dispersion. Biochemistry 11, 4120–4131. 10.1021/bi00772a015 4343790

[B7] Coque T. M. Baquero F. Canton R. (2008). Increasing prevalence of ESBL-producing Enterobacteriaceae in Europe. Euro Surveill. 13:19044. 19021958

[B8] Eftink M. R. Ghiron C. A. (1976). Exposure of tryptophanyl residues in proteins. Quantitative determination by fluorescence quenching studies. Biochemistry 15, 672–680. 10.1021/bi00648a035 1252418

[B9] Faheem M. Rehman M. T. Danishuddin M. Khan A. U. (2013). Biochemical characterization of CTX-M-15 from *Enterobacter cloacae* and designing a novel non-beta-lactam-beta-lactamase inhibitor. PLoS ONE 8:e56926. 10.1371/journal.pone.0056926 23437273 PMC3578935

[B10] Galleni M. Franceschini N. Quinting B. Fattorini L. Orefici G. Oratore A.. (1994). Use of the chromosomal class A beta-lactamase of Mycobacterium fortuitum D316 to study potentially poor substrates and inhibitory beta-lactam compounds. Antimicrob. Agents Chemother. 38, 1608–1614. 10.1128/AAC.38.7.1608 7979294 PMC284600

[B11] Gunnison J. B. Jawetz E. Coleman V. R. (1950). The effect of combinations of antibiotics on enterococci *in vitro*. J. Lab. Clin. Med. 36, 900–911. 14795023

[B12] Hasan S. Ali S. Z. Khan A. U. (2013). Novel combinations of antibiotics to inhibit extended-spectrum beta-lactamase and metallo-beta-lactamase producers *in vitro*: a synergistic approach. Future Microbiol. 8, 939–944. 10.2217/fmb.13.54 23841638

[B13] Hawkey P. M. Jones A. M. (2009). The changing epidemiology of resistance. J. Antimicrob. Chemother. 64(Suppl. 1), i3–i10. 10.1093/jac/dkp256 19675017

[B14] Jawetz E. Gunnison J. B. Speck R. S. Coleman V. R. (1951). Studies on antibiotic synergism and antagonism; the interference of chloramphenicol with the action of penicillin. AMA Arch. Intern. Med. 87, 349–359. 10.1001/archinte.1951.03810030022002 14810260

[B15] Kang J. Liu Y. Xie M. X. Li S. Jiang M. Wang Y. D. (2004). Interactions of human serum albumin with chlorogenic acid and ferulic acid. Biochim. Biophys. Acta 1674, 205–214. 10.1016/j.bbagen.2004.06.021 15374625

[B16] Karim A. Poirel L. Nagarajan S. Nordmann P. (2001). Plasmid-mediated extended-spectrum beta-lactamase (CTX-M-3 like) from India and gene association with insertion sequence ISEcp1. FEMS Microbiol. Lett. 201, 237–241. 10.1111/j.1574-6968.2001.tb10762.x 11470367

[B17] Lakowicz J. R. (1988). Principles of frequency-domain fluorescence spectroscopy and applications to cell membranes. Subcell. Biochem. 13, 89–126. 10.1007/978-1-4613-9359-7_3 2577864

[B18] Lin J. H. Cocchetto D. M. Duggan D. E. (1987). Protein binding as a primary determinant of the clinical pharmacokinetic properties of non-steroidal anti-inflammatory drugs. Clin. Pharmacokinet. 12, 402–432. 10.2165/00003088-198712060-00002 3301150

[B19] Moellering R. C. Jr. Weinberg A. N. (1971). Studies on antibiotic syngerism against enterococci. II. Effect of various antibiotics on the uptake of 14 C-labeled streptomycin by enterococci. J. Clin. Invest. 50, 2580–2584. 10.1172/JCI106758 5001959 PMC292207

[B20] O'Callaghan C. H. Morris A. Kirby S. M. Shingler A. H. (1972). Novel method for detection of beta-lactamases by using a chromogenic cephalosporin substrate. Antimicrob. Agents Chemother. 1, 283–288. 10.1128/AAC.1.4.283 4208895 PMC444209

[B21] Pérez-Llarena F. J. Kerff F. Abián O. Mallo S. Fernández M. C. Galleni M.. (2011). Distant and new mutations in CTX-M-1 beta-lactamase affect cefotaxime hydrolysis. Antimicrob. Agents Chemother. 55, 4361–4368. 10.1128/AAC.00298-11 21730121 PMC3165300

[B22] Rehman M. T. Faheem M. Khan A. U. (2015). An insight into the biophysical characterization of different states of cefotaxime hydrolyzing beta-lactamase 15 (CTX-M-15). J. Biomol. Struct. Dyn. 33, 625–3821. 10.1080/07391102.2014.899925 24650131

[B23] Rehman M. T. Shamsi H. Khan A. U. (2014). Insight into the binding mechanism of imipenem to human serum albumin by spectroscopic and computational approaches. Mol. Pharm. 11, 1785–1797. 10.1021/mp500116c 24745377

[B24] Tiwari V. Moganty R. (2014). Conformational stability of OXA-51 b -lactamase explains its role in carbapenem resistance of *Acinetobacter baumannii*. J. Biomol. Struct. Dyn. 32, 1406–1420. 10.1080/07391102.2013.819789 23879430

