# Supplementary material for: A Mechanism of Synergistic Effect of Streptomycin and Cefotaxime on CTX-M-15 Type β-lactamase Producing Strain of E. cloacae: A First Report
Source: Front Microbiol. 2016 Dec 15;7:2007. doi: 10.3389/fmicb.2016.02007 (PMC5156679; doi:10.3389/fmicb.2016.02007)
Supplement: Supplementary file 1 [file Image1.PDF]

(Supplementary figures)

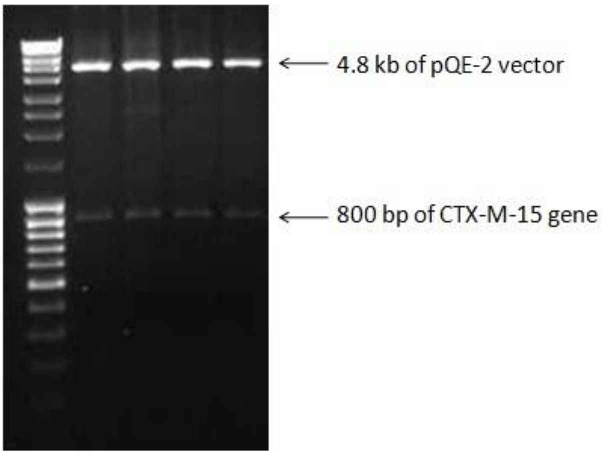

Figure S1: Agarose gel showing digestion product of pQE-2 vector harbouring *bla*$_{\text{CTX-M-15}}$ by NdeI and HindIII. The brighter band in four lanes is of vector backbone of 4.8 kb and the faint band is of CTX-M-15 gene of approximately 800 bp.

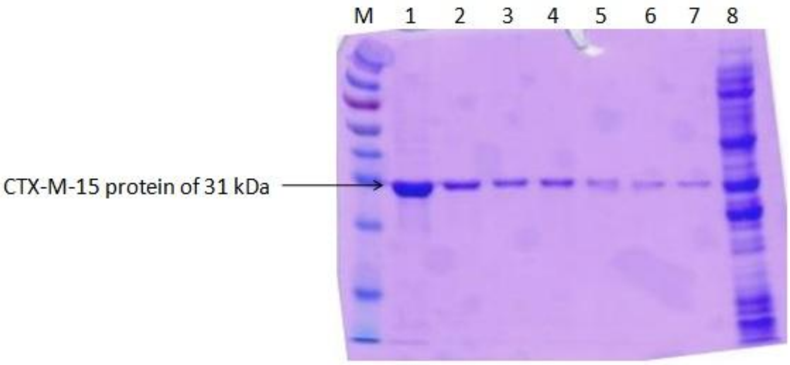

Figure S2: SDS polyacrylamide gel showing lane 1, 2, 3, 4, 5, 6 & 7of soluble protein fractions from *E. coli* BL21 cell lysates harbouring CTX-M-15 gene grown at 16°C for 15 hours & induced with 0.2 mM IPTG. Lane 8 showing insoluble protein fraction. Lane M is prestained protein ladder.

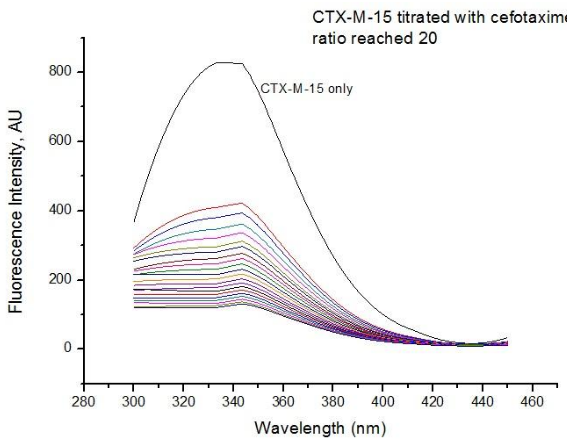

A

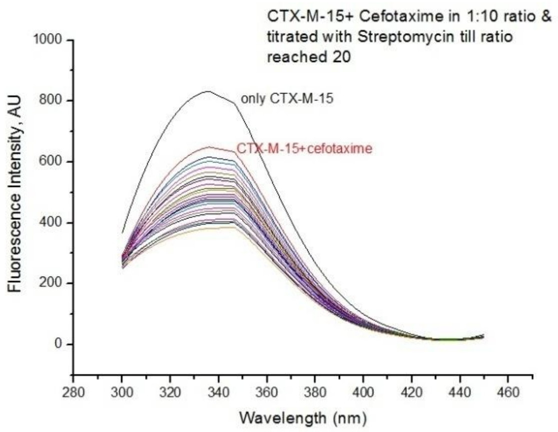

B

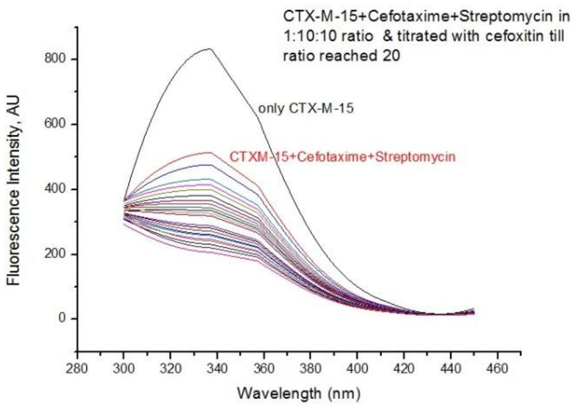

C

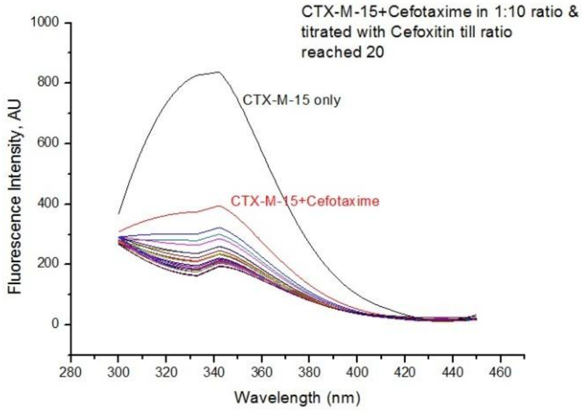

D

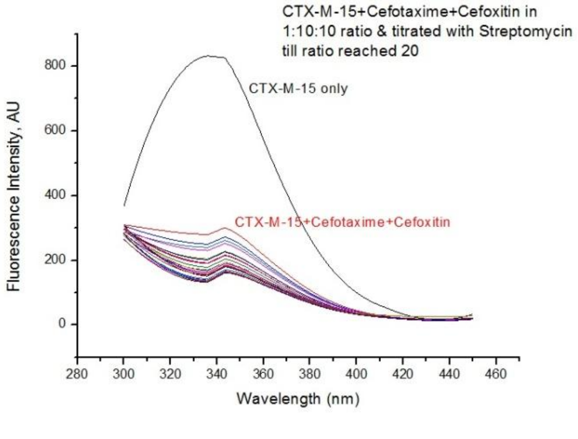

E

Figure S3: Drug-induced fluorescence quenching of CTX-M-15. The concentration of CTX-M-15 was 2 μM, the concentration of the bounded drug was 20 μM and the concentration of drug to be titrated was varied from 0 to 40 μM in a successive increment of 2 μM. Figure shows A, B, C, D & E plots and the decrease in their relative fluorescence intensity by drug binding. Plot A shows binding of cefotaxime to enzyme, B shows binding of streptomycin to cefotaxime bounded enzyme, C shows binding of cefoxitin to cefotaxime and streptomycin bounded enzyme, D shows binding of cefoxitin to cefotaxime bounded enzyme and E corresponds to binding of streptomycin to cefotaxime and cefoxitin bounded enzyme. The intrinsic fluorescence of the protein was measured in 50 mM sodium phosphate buffer, pH 7.4 at 298 K upon excitation at 295 nm.

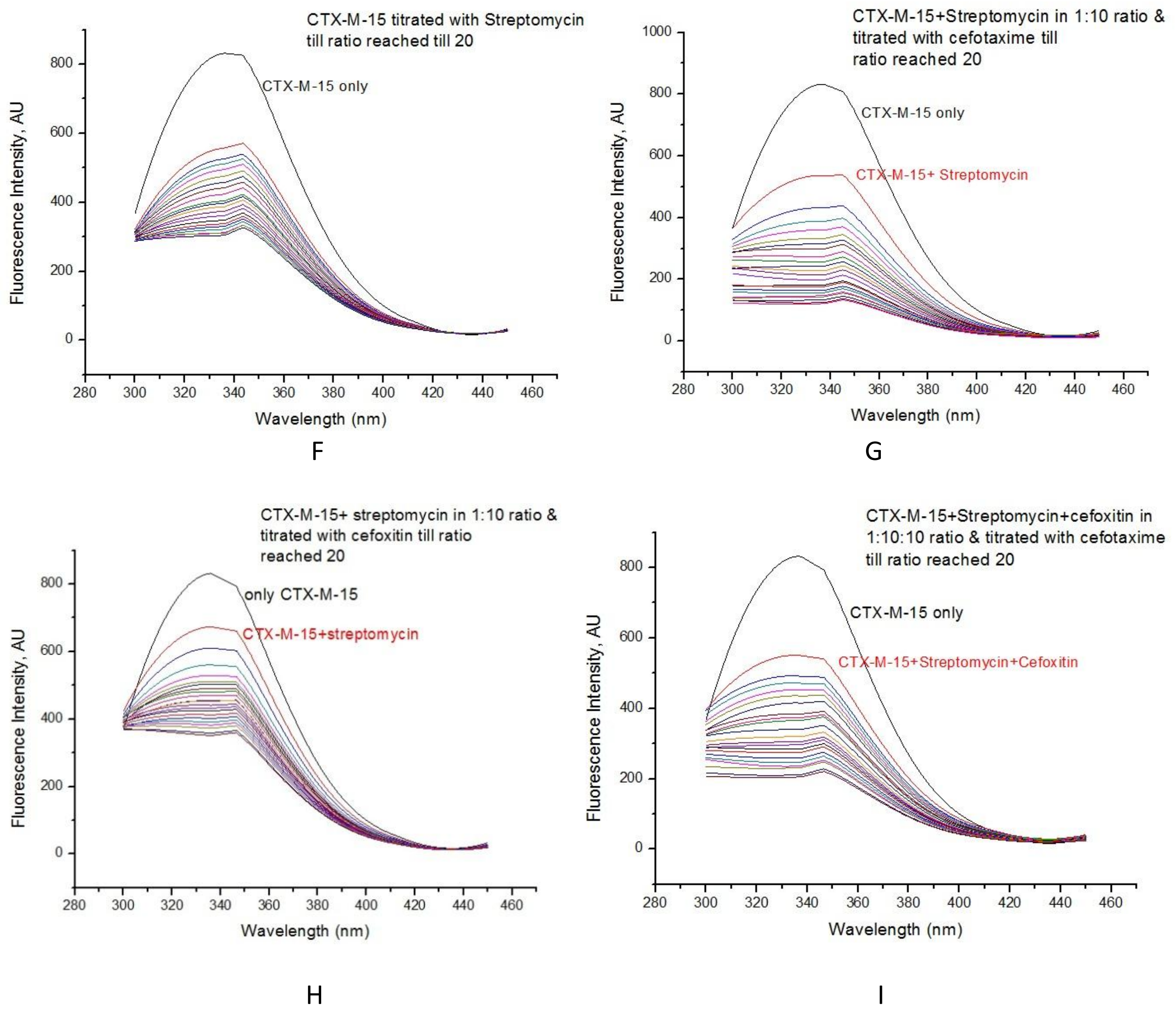

Figure S4: Drug-induced fluorescence quenching of CTX-M-15. The concentration of CTX-M-15 was 2 μM, the concentration of the bounded drug was 20 μM and the concentration of drug to be titrated was varied from 0 to 40 μM in a successive increment of 2 μM. Figure shows F, G, H & I plots and the decrease in their relative fluorescence intensity by drug binding. Plot F shows binding of streptomycin to enzyme, G shows binding of cefotaxime to streptomycin bounded enzyme, H shows binding of cefoxitin to streptomycin bounded enzyme and I shows binding of cefotaxime to streptomycin and cefoxitin bounded enzyme. The intrinsic fluorescence of the protein was measured in 50 mM sodium phosphate buffer, pH 7.4 at 298 K upon excitation at 295 nm.
